# KAF156 Is an Antimalarial Clinical Candidate with Potential for Use in Prophylaxis, Treatment, and Prevention of Disease Transmission

**DOI:** 10.1128/AAC.02727-13

**Published:** 2014-09

**Authors:** Kelli L. Kuhen, Arnab K. Chatterjee, Matthias Rottmann, Kerstin Gagaring, Rachel Borboa, Jennifer Buenviaje, Zhong Chen, Carolyn Francek, Tao Wu, Advait Nagle, S. Whitney Barnes, David Plouffe, Marcus C. S. Lee, David A. Fidock, Wouter Graumans, Marga van de Vegte-Bolmer, Geert J. van Gemert, Grennady Wirjanata, Boni Sebayang, Jutta Marfurt, Bruce Russell, Rossarin Suwanarusk, Ric N. Price, Francois Nosten, Anchalee Tungtaeng, Montip Gettayacamin, Jetsumon Sattabongkot, Jennifer Taylor, John R. Walker, David Tully, Kailash P. Patra, Erika L. Flannery, Joseph M. Vinetz, Laurent Renia, Robert W. Sauerwein, Elizabeth A. Winzeler, Richard J. Glynne, Thierry T. Diagana

**Affiliations:** a Genomics Institute of the Novartis Research Foundation, San Diego, California, USA; b Swiss Tropical and Public Health Institute, Parasite Chemotherapy, Basel, Switzerland; c University of Basel, Basel, Switzerland; d Department of Microbiology and Immunology, Columbia University College of Physicians and Surgeons, New York, New York, USA; e Division of Infectious Diseases, Department of Medicine, Columbia University College of Physicians and Surgeons, New York, New York, USA; f Radboud University Nijmegen Medical Center, Medical Microbiology Department, Nijmegen, The Netherlands; g Global Health Division, Menzies School of Health Research, Charles Darwin University, Darwin, Australia; h Eijkman Institute for Molecular Biology, Jakarta, Indonesia; i Laboratory of Malaria Immunobiology, Singapore Immunology Network, Agency for Science Technology and Research, Biopolis, Singapore; j Shoklo Malaria Research Unit, Mae Sot, Tak, Thailand; k Faculty of Tropical Medicine, Mahidol University, Bangkok, Thailand; l Centre for Tropical Medicine, Nuffield Department of Clinical Medicine, University of Oxford, Oxford, United Kingdom; m Department of Veterinary Medicine, U.S. Army Medical Component, Armed Forces Research Institute of Medical Sciences, Bangkok, Thailand; n Entomology Department, AFRIMS, Bangkok, Thailand; o Division of Infectious Diseases, Department of Medicine, University of California, San Diego, La Jolla, California, USA; p Division of Pharmacology and Drug Discovery, University of California, San Diego, School of Medicine, La Jolla, California, USA; q Novartis Institute for Tropical Diseases, Singapore

## Abstract

Renewed global efforts toward malaria eradication have highlighted the need for novel antimalarial agents with activity against multiple stages of the parasite life cycle. We have previously reported the discovery of a novel class of antimalarial compounds in the imidazolopiperazine series that have activity in the prevention and treatment of blood stage infection in a mouse model of malaria. Consistent with the previously reported activity profile of this series, the clinical candidate KAF156 shows blood schizonticidal activity with 50% inhibitory concentrations of 6 to 17.4 nM against P. falciparum drug-sensitive and drug-resistant strains, as well as potent therapeutic activity in a mouse models of malaria with 50, 90, and 99% effective doses of 0.6, 0.9, and 1.4 mg/kg, respectively. When administered prophylactically in a sporozoite challenge mouse model, KAF156 is completely protective as a single oral dose of 10 mg/kg. Finally, KAF156 displays potent Plasmodium transmission blocking activities both *in vitro* and *in vivo*. Collectively, our data suggest that KAF156, currently under evaluation in clinical trials, has the potential to treat, prevent, and block the transmission of malaria.

## INTRODUCTION

Widespread resistance to most antimalarial drug classes has led to a global adoption of artemisinin-based combination therapies (ACTs) as first-line therapies (1, 2). However, recent reports of delayed rates of parasite clearance after administration of artemisinin derivatives raise concerns that ACTs efficacy might soon be compromised (3–7). In addition to having to overcome artemisinin resistance, next-generation antimalarial drugs are also expected to target multiple stages of the parasite life cycle. The Malaria Eradication Agenda Initiative has defined the ideal antimalarial drug profile as a Single Encounter Radical Cure and Prophylaxis (SERCaP) therapy that could be used in mass administration programs (8). Upon administration of a single dose, SERCaP therapy should eliminate all asexual and sexual (mature gametocyte) blood stages of the parasite, as well as the hepatic forms, thereby providing a combined therapeutic radical cure, disease-transmission blocking and prophylactic activities (9, 10). There are currently very few antimalarial drugs with the pharmacological profile required for these next-generation therapies (11).

As an initial step toward development of a next-generation antimalarial therapy, we have previously described the imidazolopiperazines, a novel class of antimalarial drugs class with potent blood-stage (12) and liver-stage (13) activity. Here, we describe the preclinical antimalarial profile of the drug candidate, KAF156, which emerged from an extensive lead-optimization program of the imidazolopiperazine class (14). Compared to other compounds in its class, KAF156 was selected for its overall superior profile which balances the adequate physicochemical properties required for oral tablet formulation and excellent antimalarial properties. Like other imidazolopiperazine compounds, KAF156 has potent activity on blood and hepatic stage parasites which translates into therapeutic and prophylactic activity in mouse models of infection. Furthermore, KAF156 displays cidal activity against mature Plasmodium falciparum gametocytes and thus blocks parasite transmission to Anopheles mosquitoes. Taken together, our data suggest that KAF156 combined with other antimalarial drugs could be used in malaria elimination campaigns to prevent infection, treat acute disease and reduce transmission of the parasite.

## MATERIALS AND METHODS

### Maintenance of P. falciparum cultures.

P. falciparum parasites were cultured in O^+^ red blood cells in RPMI 1640 media (without phenol red) containing l-glutamine and supplemented with 50 μg of gentamicin/ml, 14 mg of hypoxanthine/liter, 38.4 mM HEPES, 0.2% sodium bicarbonate, 0.2% glucose (pH 7.2), 5% human serum, and 0.25% Albumax II. Cultures were maintained at 5% hematocrit at a parasitemia of 1 to 10%, with daily media changes (15). Fresh blood was drawn at least every 2 weeks, and cultures were maintained under 96% nitrogen, 3% carbon dioxide, and 1% oxygen at 37°C.

### Antimalarial proliferation inhibition assay (384-well plate format).

A 20-μl portion of screening medium (culturing medium without human serum but supplemented with 0.5% Albumax II) was dispensed via a MicroFlo (BioTek) liquid dispenser into 384-well, black, clear-bottom assay plates (μClear GNF custom plates; Griener Bio-One). Then, 50-nl portions of compounds in a dose-response format were transferred by using a PlateMate Plus (Matrix) or the PinTool (GNF Systems). Each parasite strain was diluted to 0.3% parasitemia and 4.2% hematocrit and dispensed (30 μl) into prewarmed assay plates by using the MicroFlo liquid dispenser. The final parasitemia and hematocrit were 0.3 and 2.5%, respectively. Assay plates were incubated at 37°C for 72 h under 96% nitrogen, 3% carbon dioxide, and 1% oxygen. After 72 h, a prepared mixture of lysis buffer (5 mM EDTA, 1.6% Triton X-100, 20 mM Tris-HCl, 0.16% saponin) in water and 0.1% SYBR green detection reagent was dispensed at 10 μl per well using the MicroFlo. Cultures were incubated for an additional 24 h at 25°C before measuring the fluorescence intensity using an Envision plate reader (Perkin-Elmer) with a 505 dichroic mirror. The excitation and emission filters were 485 and 530 nm, respectively. The data were normalized based on the maximum fluorescence signal values for dimethyl sulfoxide (DMSO)-treated wells (no inhibition by compound) and the minimum fluorescence signal values for wells containing the highest concentration of inhibitor control compounds. The 50% inhibitory concentrations (IC_50_s) were obtained by using a curve-fitting model with standard logistic regression.

### Clinical isolate schizont maturation assay.

Field isolates were collected from the Mae Sot region of Tak Province (Thailand) in 2009 (10 P. vivax and 13 P. falciparum) and Timika, Southern Papua (Indonesia), in 2011 (20 P. vivax and 26 P. falciparum). All samples were from patients with acute malaria (with a monospecies parasitemia of 2,000 to 10,000 parasites/μl) attending outpatient clinics. After written consent was obtained, blood samples were collected by venipuncture into heparinized tubes and processed within 5 h of collection. Ethical approval for this project was provided by the Human Research Ethics Committee, Menzies School of Health Research, Darwin, Australia (HREC 2010-1396); the Eijkman Institute Research Ethics Commission, Jakarta, Indonesia (EIREC-47); the University of Oxford, Centre for Clinical Vaccinology and Tropical Medicine, United Kingdom (XTREC 027-025); and the Ethics Committee of the Faculty of Tropical Medicine, Mahidol University, Bangkok, Thailand (MUTM 2008-215). Samples with >80% early rings were chosen for drug sensitivity testing. After the platelets and leukocytes were removed, drug sensitivity and stage-specific activity were tested as previously described (16, 17). Drug plate quality was assured by running schizont maturation assays with the chloroquine-resistant strain K1 and the chloroquine-sensitive strain FC27. Dose-response curves and IC_50_ values were calculated by fitting the data to a sigmoidal inhibitory *E*_max_ pharmacodynamic model using WinNonLin (v4.1; Pharsight Corp.). The median IC_50_s were compared nonparametrically using the Kruskal-Wallis test. Statistical analyses and graphics were carried out using GraphPad Prism 5 software (version 5).

### P. falciparum gametocyte maturation.

NF54 cultures were initiated in a tipper system in a suspension of 5% group 0 red cells in culture medium with an asexual parasite density of 0.5%, and the medium was replaced daily (18–20). Asexual parasites and gametocytes were monitored regularly using Giemsa-stained blood films. At day 8, all cultures containing 3 to 4% asexual parasite-infected red blood cells, 2.5% stage II gametocytes, and 2.4% stage III/IV gametocyte stages (total gametocytemia of 4.9%) were pooled. Purification methods were not used for the removal of asexual parasites to avoid potential negative effects on gametocyte maturation and infectivity. Compound was added from days 8 to 12 with medium replacement twice daily and replaced by control medium at 48 h before the standard membrane feeding assay (SMFA). The number of mature gametocytes was determined at day 13, as well as the capacity of male gametocytes to exflagellate after temperature drop and addition of fetal calf serum (21). Cultures with exflagellating gametocytes and/or mature gametocytes were fed in the SMFA to mosquitoes on day 14.

### Transmission blocking assays.

The SMFA was used to test the potential effects of compounds or drugs on parasite sporogony in the mosquito as previously described (22, 23). Briefly, 14-day-old cultures of strain NF54 showing 0.3 to 0.5% mature gametocytes were first checked for their quality and potential to form oocysts in a preliminary test feed to anopheles mosquitoes. When ookinetes were seen 22 h after the feeding, parasite culture material was used to test the potential effects of compounds on sporogony. First, 300 μl of culture material was added to 180 μl of washed packed cells and then centrifuged for 20 s. Next, after removal of the supernatant, 150 μl of human control serum with or without test compound was added to the pellet. Each suspension was immediately injected into an individual membrane-covered minifeeder and 20 3- to 5-day-old Anopheles stephensi mosquitoes were allowed to feed for 10 to 15 min. Six days after feeding, 20 mosquitoes per feeder were dissected. Absolute numbers of oocysts were counted by light microscopy after staining the mosquito stomach with 2% mercurochrome. Oocyst numbers were calculated as the arithmetic mean of the number of oocysts of 20 dissected mosquitoes and represent the transmission-reducing activity of the compounds tested. The *in vivo* assessment of transmission blocking activity was performed with rodent parasites as follows. This study was conducted in accordance with U.S. animal welfare regulations under a protocol approved by the University of California at San Diego Institutional Animal Care and Use Committee. Mice were infected with 5 × 10^5^ P. berghei ANKA 676m1cl1 parasites on day 0 via an intraperitoneal injection. The mice were then dosed with 100 mg of KAF156/kg (formulated in 0.5% [wt/vol] methylcellulose [Sigma-Aldrich, catalog no. M0262] and 0.5% [vol/vol] Tween 80) or vehicle control orally on day 5 when the parasitemia reached 5 to 6%. Feeds were completed 24 h later on day 6. Oocysts were counted by light microscopy after the mosquito stomachs were stained with 2% mercurochrome.

### P. yoelii liver-stage antimalarial assay.

This assay has previously been described (13). In brief, P. yoelii (17XNL) sporozoites were obtained after salivary gland dissection of infected A. stephensi mosquitoes supplied by the New York University Insectary. Dissected salivary glands were homogenized in a glass tissue grinder, filtered twice through nylon cell strainers (40-μm pore size; BD Falcon), and counted using a hemocytometer. Then, 7.5 × 10^3^ HepG2-A16-CD81^EGFP^ cells in 50 μl of medium (1.5 × 10^5^ cells/ml) were seeded in 384-well plates (Aurora 384 IQ-EB black/clear plates) 20 to 26 h prior to the actual infection. Two hours prior to infection, 50 nl of compound in DMSO (0.1% final DMSO concentration per well) were transferred with a PinTool (GNF Systems) into the assay plates (10 μM final concentration). Atovaquone, an exo-erythrocytic schizonticidal approved drug (10 μM) and 0.1% DMSO were used as positive and negative controls, respectively. The HepG2-A16-CD81^EGFP^ cells were then infected with 8 × 10^3^ sporozoites per well, and the plates were spun down at 650 × *g*. After infection and 1 h of incubation at 37°C, the cultures were washed, new media and compound were added, and the cultures were further incubated with 5-fold-increased concentrations of penicillin-streptomycin for 48 h at 37°C. Infected cells were quantified by immunofluorescence. About 8,000 sporozoites were used to infect one well, and the infected HepG2-A16-CD81^EGFP^ cells were exposed for 48 h to compounds at a 10 μM concentration dissolved in DMSO. About half of the total area of a well was imaged, which on average covered about 50 infected cells.

### *In vivo* mouse causal prophylaxis efficacy assay.

These experiments were conducted at the USAMC-AFRIMS facility (accredited by the Association for Assessment and Accreditation of Laboratory Animal Care International) in Bangkok, Thailand. In brief, mice (five per experimental group) received test compound on day 0, 2 h before parasite inoculation. Control animals received the same amount of vehicle without drug. Atovaquone, an approved prophylactic antimalarial drug, was used as a positive control. We have previously established that atovaquone, when administered as a single oral dose of 2.5 mg/kg, is fully protective in this model (unpublished data). On day 0, all ICR (outbred stock) mice were infected by a standard 0.1-ml dose of 10^5^ P. berghei sporozoites by intravenous inoculation. Blood smear samples were obtained on days 4, 5, 6, 7, 10, 15, 21, and 31 postinoculation. Mice were observed twice daily for clinical signs and mortality. On day 7 and afterward, animals with parasitemia exceeding 5% were euthanized. In a dose-ranging study, KAF156 was dosed orally in a suspension formulation of 0.5%, (wt/vol) methylcellulose, and 1% (wt/vol) Solutol HS15 at 1, 5, 10, or 15 mg/kg. The prophylactic activity was monitored through blood smear analysis and is expressed in terms of mouse survival over 30 days.

### *In vivo* mouse therapeutic efficacy assay.

All *in vivo* efficacy studies were approved by the veterinary authorities of the Canton Basel-Stadt. The *in vivo* antimalarial activity was usually assessed for groups of five female NMRI mice (20 to 22 g) intravenously infected on day 0 with 2 × 10^7^ erythrocytes parasitized with P. berghei green fluorescent protein (GFP)-expressing parasites (PbGFPCON, kindly donated by A. P. Waters and C. J. Janse, Glasgow and Leiden Universities) (24, 25) (for reference compounds, see Table 1 and also Table S2 in the supplemental material) or the P. berghei ANKA reference strain (for all other studies). From historical data, untreated control mice died typically between days 6 and 7 postinfection, but in these studies all animals showing unabated parasitemia or malaria symptoms were humanely euthanized on day 4 with CO_2_. Experimental compounds were formulated in 7% (vol/vol) Tween 80–3% (vol/vol) ethanol, 10% ethanol (vol/vol), 30% (wt/vol) PEG400, 60% (wt/vol) Vit E TPGS (Eastman), or 5% (wt/vol) Solutol HS15 (BASF) as indicated. Compounds were administered orally in a volume of 10 ml/kg as a single dose (24 h postinfection), as three consecutive daily doses (24, 48, and 72 h postinfection), or as four consecutive doses (6, 24, 48, and 72 h postinfection). With the single-dose regimen we determined the parasitemia at 72 h postinfection, and for the triple- and quadruple-dose regimens we determined the parasitemia at 96 h postinfection using standard flow cytometry techniques or standard microscopy (25). The activity was calculated as the difference between the mean percent parasitemia for the control and treated groups expressed as a percentage of the control group. The survival time in days was also recorded up to 30 days after infection. A compound was considered curative if the animal survived to day 30 after infection with no detectable parasites.

**TABLE 1:** *In vivo* efficacy of KAF156 in a P. berghei rodent malaria model^*a*^

Dose(s) (mg/kg)	Compound^*b*^	Mean ± SD		Cure (%)^*d*^
		Activity (%)	Mouse survival (days)^*c*^	
1 × 30	Chloroquine*	99.9 ± 0.07	9.6 ± 0.8	0
	Mefloquine*	99.6 ± 0.5	21.8 ± 2.1	0
	Artesunate*	92.4 ± 2.9	9.0 ± 1.2	0
	KAF156†	99.9 ± 0.006	16.7 ± 2.8	0
1 × 100	Chloroquine*	99.9	12.7	0
	Artesunate*	99.9 ± 0.08	8.8 ± 0.8	0
	KAF156†	99.9 ± 0.008	23.4 ± 4.3	10
3 × 30	Chloroquine‡	99.9 ± 0.02	14.0 ± 0.0	0
	Mefloquine‡	98.6 ± 0.3	18.8 ± 1.3	0
	Artesunate‡	99.0 ± 0.3	11.8 ± 3.3	0
3 × 50	KAF156†	>99.99	29.4 ± 0.8	40
4 × 100	KAF156†	>99.99	29.8 ± 0.6	90

a A comparison of single- and multiple-dose efficacies with standard antimalarials or KAF156 was performed. For single-dose efficacy, the mice were dosed 24 h after infection. The percent parasitemia was measured 72 h after infection, and mouse survival was monitored. For multiple-dose efficacy, the mice were dosed daily for 3 days (24, 48, and 72 h after infection) or 4 days (6, 24, 48, and 72 h after infection [see column 1]) at the indicated doses. The percent parasitemia was measured 96 h after infection, and mouse survival was monitored. GFP-ANKA was used in the reference study with chloroquine, mefloquine, and artesunate, and parasitemia was determined by fluorescence-activated cell sorting analysis. No significant differences were observed with GFP-ANKA and ANKA strains for these compounds. Data are means calculated from two independent experiments, with *n* = 5 mice for each experiment.

b *, Ethanol-Tween 80-water (3/7/90); †, 5% Solutol HS15; ‡, 10% ethanol, 30% PEG400, 60% Vit E TPGS.

c The survival of control animals was 6 to 7 days.

d Cure, no parasites present at day 30.

### *In vitro* resistance selection.

A clonal population of P. falciparum strain Dd2 was used to initiate three independent parasite cultures under the initial selection pressure of 1.8 nM KAF156 (flask/strain 1, 2, and 3). Parasitemia was monitored daily, and the compound concentration was increased 2-fold when parasitemia reached ≥3%. After 1 month of selection, each culture was split into two cultures (culture 1, 1A and 1B; culture 2, 2A and 2B; and culture 3, 3A and 3B) in an attempt to accelerate resistance development in each pair by increasing the concentration >2-fold (“B” strains) generating ultimately six resistant strains after 4 months of continuous culture in increasing concentrations of KAF156. For each of the six resistant strains, the single-nucleotide polymorphisms (SNPs) were detected by either whole genome sequencing (Illumina technology) or capillary sequencing (see below).

The susceptibility of each resistant strain to KAF156, GNF707, and GNF452 (the GNF707 and GNF452 chemical structures and synthesis are described elsewhere [13]) was determined, and the fold shift in efficacy (i.e., the phenotypic susceptibility shift) was calculated.

For determination of the resistance frequency, cloned lines of Dd2 (1pa) and FCR3 were cultured in complete RPMI 1640 medium (0.5% Albumax) in various volumes (2 to 320 ml). Parasites were pressured with ∼3× IC_50_ in a single-step selection and cultured for either 60 days or until resistant parasites emerged under selection. Prior to selection, the starting parasite cultures were expanded to ∼1.5 liters in multiple flasks, pooled, and distributed according to the appropriate starting inoculum. Predominantly ring-stage cultures were exposed to drug, and cultures were fed daily for 6 days and every other day thereafter until the emergence of parasites.

### Drug-resistant mutants genomic analysis.

The design of PCR primers to amplify a single gene we named P. falciparum Cyclic Amine Resistance Locus (*pfcarl*; PlasmoDB ID PFC0970w) for Sanger sequencing was problematic due to the high percentage of A+T nucleotides in this gene. We therefore designed long PCR primers around the gene, avoiding high A+T regions, to amplify two overlapping fragments spanning ∼5.9 kb total (primer sequences below). Sequencing libraries were made from these fragments using Nextera library preparation kits (Illumina, San Diego, CA). Paired 60-bp reads were generated on an Illumina GAIIx instrument. Reads were aligned to the P. falciparum strain Dd2 using SOAP (26). Nucleotide changes from the reference sequence were called where at least six separate reads called the alternate letter, and the sum of the Illumina quality scores of the alternate letter from all reads containing that letter were 5× the sum of the Illumina quality score of the reference letter in all of the reads that contained that letter. A new reference sequence was generated with these SNP changes, and the reads were aligned with SOAP against the improved genomic sequence; new SNPs were discovered, and this process was iterated nine times. The long PCR primer sequences were as follows: pfc970w_long1F, TTTGTCTTTTCTAGTTATAATGATTT; pfc970w_long1R, GATCTGTAGTAATAACTGATTGTGGTGAAT; pfc970w_long2F, TTTTGTCTTTTCTAGTTATAATGATTTTTTAAAAAACTAAAAGAAGCAC; and pfc970w_long2R, GTCAAAAGATCTGTAGTAATAACTGATTGTGGTGAAT.

## RESULTS

### KAF156 potently inhibits blood stages of Plasmodium species.

**FIG 1:**
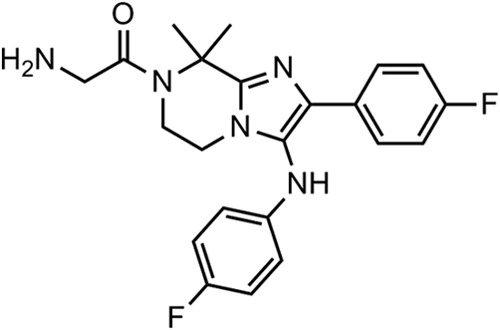
Chemical structure of KAF156.

It was previously shown that KAF156 (Fig. 1) has low nanomolar potency in inhibiting the growth of laboratory-adapted P. falciparum strains cultured in human erythrocytes (6 to 17 nM IC_50_) using a 72-h SYBR green proliferation assay (14). This activity range is maintained over a broad panel of strains resistant to one or more current antimalarial drugs (see Table S1 in the supplemental material).

**FIG 2:**
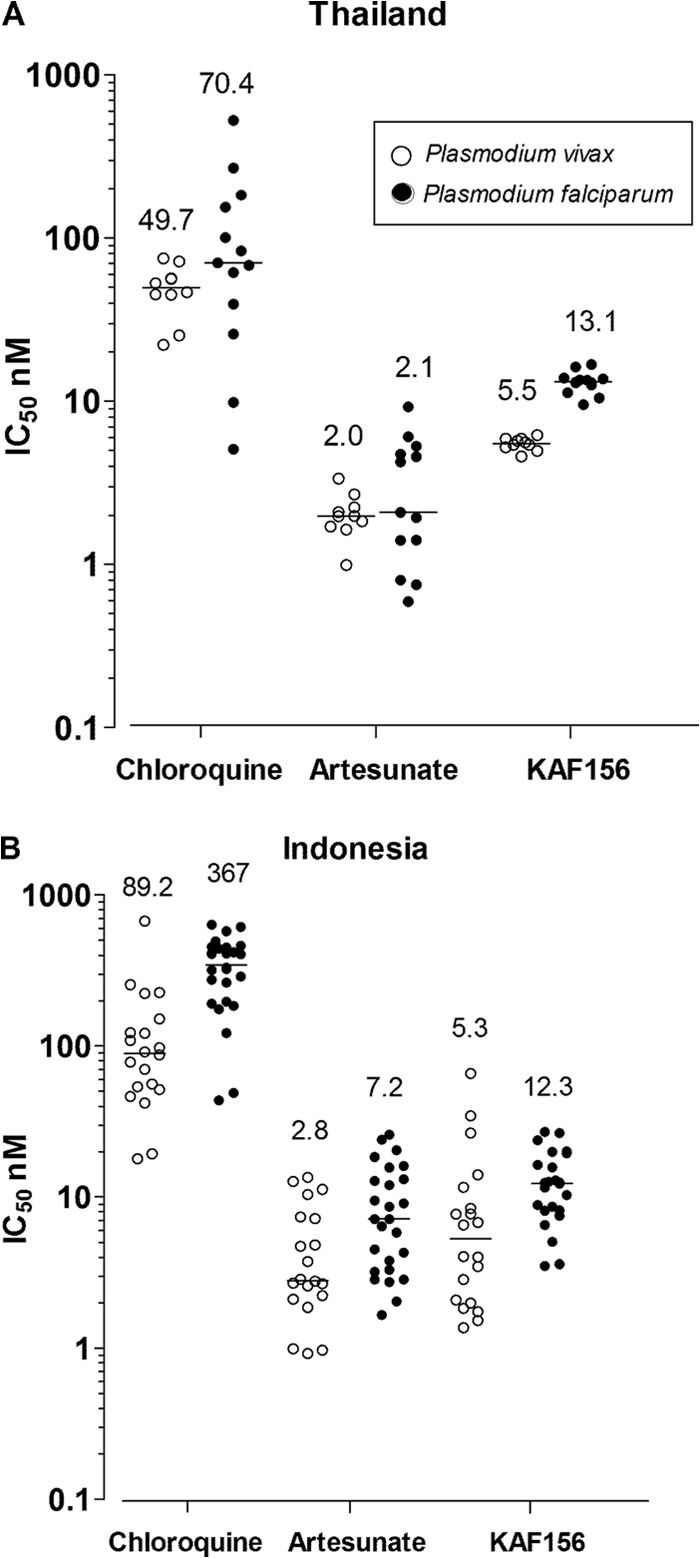
Drug sensitivity of P. vivax (○) and P. falciparum (●) clinical isolates to chloroquine, artesunate, and KAF156. An *ex vivo* schizont maturation assay was used to measure the drug sensitivity of clinical isolates in Thailandfor P. vivax (*n* = 10) and P. falciparum (*n* = 13) isolates (A) and in Indonesia for P. vivax (*n* = 20) and P. falciparum (*n* = 26) (B). The horizontal bar represents the median IC_50_, and its numerical value is indicated above (nM).

We extended these studies to clinical isolates of P. vivax and P. falciparum collected from malaria patients on the Thai-Myanmar border and Papua Indonesia, where multidrug resistance has been reported in both P. falciparum and P. vivax (27–30). An *ex vivo* schizont maturation assay (16) was used to measure activity on asexual development. Across the two Thai and Indonesian sites the overall median IC_50_ values were 12.6 nM (range, 3.5 to 27.1 nM) against P. falciparum and 5.5 nM (range, 1.4 to 65.8 nM) against P. vivax (Fig. 2). At both sites, drug susceptibility to KAF156 was significantly greater than that for chloroquine but less that than for artesunate (*P* < 0.001 in Kruskal-Wallis analysis).

We have previously reported therapeutic activity for KAF156 in a malaria mouse model (14), using the P. berghei GFP expressing ANKA-strain (25) and, for reasons that remain unclear, we did not observe a dose-dependent increase in mouse survival with this parasite strain. In contrast, when the original P. berghei ANKA strain (without the GFP transgene) was used, KAF156 achieved sterilizing activity upon administration of four daily 100-mg/kg doses in 9 of 10 mice, with no detectable parasites 30 days after infection (Table 1). This does not appear to be driven by an intrinsically higher drug sensitivity of the ANKA parasite, since oral dosing of KAF156 reduced blood parasitemia with potency (50% effective dose [ED_50_] = 0.6 mg/kg, ED_90_ = 0.9 mg/kg, and ED_99_ = 1.4 mg/kg [reported in Table S2 in the supplemental material]) comparable to what we have previously observed with the GFP-ANKA strain (14). Nonetheless, our data collectively show that KAF156 is at least as effective as some of the current antimalarial drugs for the treatment of an acute blood-stage malaria infection.

### Generation and characterization of KAF156 drug-resistant mutants *in vitro*.

The development of drug resistance has historically rapidly limited the efficacy and therefore the use of many approved antimalarial drugs. To assess the potential for developing resistance to imidazolopiperazines and using the stepwise drug resistance selection method previously reported (13), we selected P. falciparum clones for resistance to KAF156 and some related compounds (Table 2). Consistent with our previous findings, targeted sequencing analysis of these clones showed that all resistant lines had acquired SNPs in a single gene we named *pfcarl* (PlasmoDB ID PFC0970w), encoding an uncharacterized protein conserved across Plasmodium species with seven predicted transmembrane regions (13). Unlike the earlier derivatives GNF707 and GNF452, KAF156 generally displayed potent activity against almost all of the drug resistant clones (Table 2). The only exception was the clone bearing the *pfcarl* S1076I mutation, to which KAF156 proved considerably less potent.

**TABLE 2:** *In vitro* resistance mechanism of KAF156^*a*^

Selection agent	Culture	SNP(s)^*b*^	Mean IC_50_ (nM) ± SD		
			GNF707	GNF452	KAF156
DMSO		None (Dd2, WT)	8.1 ± 0.9	7.6 ± 2.0	1.8 ± 0.6
GNF707	1R	V1254F	170	49	2.0
	2R	L830V	150	61	2.0
	3R	E834D	1,300	360	36
GNF452	1R	M1069I, L830V	3,600	1,200	97
	3R	S1076I, L830V	2,600	7,300	3,600
KAF156	1AR	Q821H, S1076R	5,300	1,900	10
	1BR	P822T, S1076R	>10,000	>10,000	73
	2BR	S1076I	3,000	>10,000	1,600
	3AR	E834D	6,900	1,800	24
	3BR	E834D	5,000	1,600	16

a Five independent cultures of P. falciparum strain Dd2 were cultured in the presence of increasing concentrations of KAF156 over 4 months and, in a similar manner, three independent cultures were pressured with GNF707, and two independent cultures were pressured with GNF452. Parasitemia was monitored daily, and the compound concentration was increased 2-fold when the parasitemia reached ≥3%. For each of the resistant strains, the SNPs detected and the antimalarial activities (IC_50_) are as indicated for KAF156, GNF707, and GNF452. Data are presented to two significant figures as the median value from two to four independent experiments; the IC_50_s were consistent within 1.5-fold across experiments.

b SNPs, single-nucleotide polymorphisms. SNPs were confirmed by capillary sequencing of *pfcarl* (PFC0970w).

Notably, whereas standard drugs remain potently active (see Table S3 in the supplemental material), earlier imidazolopiperazine analogs were found generally to be inactive against the KAF156-resistant strains. In one of these KAF156-resistant clones, we identified two novel SNPs (see Fig. S1 in the supplemental material), namely, S1076R and P822T, that in combination shifted the potency of KAF156 to an IC_50_ of 73 nM (∼40-fold higher than against the sensitive parental line). However, of all the SNPs identified, substitution of the serine at position 1076 with an isoleucine appears to be the most detrimental mutation, yielding a drug sensitivity shift to KAF156 of >700-fold.

In order to estimate the frequency of drug resistance mutations in P. falciparum, we used the method of minimal inoculum for resistance that is an indirect measurement of the probability of a resistant genotype to occur (31). We selected for spontaneous mutants emerging from cultures of both Dd2 and FCR3 strains by exposing a range of starting inocula (1 × 10^6^ to 4 × 10^9^ parasites) to constant drug pressure at ∼3× IC_50_ for 60 days. Under these conditions, we observed drug-resistant parasites emerging only from Dd2 cultures containing more than 10^8^ parasites (see Table S4 in the supplemental material). Again, targeted sequencing analysis revealed that all KAF156-resistant clones with a significant shift in potency carried nonsynonymous SNPs in the *pfcarl* gene (I1139K or Q821H). Collectively, our data suggest that *in vitro* selection of resistant mutations to KAF156 arise in the *pfcarl* gene with a frequency of ∼1 per 10^8^ parasites with the Dd2 P. falciparum strain.

### KAF156 has potent activity on liver-stage parasites *in vitro* and has prophylactic activity *in vivo*.

We have previously shown that imidazolopiperazines inhibit the growth of the exo-erythrocytic forms in an *in vitro* assay using CD81-expressing HepG2 hepatoma cells infected with rodent P. yoelii liver-stage parasites (13). Similarly, KAF156 displayed potent activity in this assay with an IC_50_ of 2 nM against intrahepatic schizonts.

**FIG 3:**
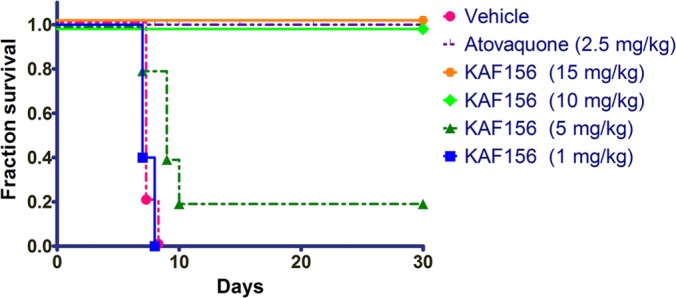
KAF156 is fully protective in a causal prophylactic mouse model of malaria. Mice (*n* = 5) were infected with P. berghei sporozoites intravenously on day 0 and administered a single dose of compound 2 h before parasite inoculation. Mice were monitored over 30 days for parasitemia. Vehicle used for compound administration was a suspension formulation of 0.5% (wt/vol) methylcellulose and 1% (wt/vol) Solutol HS15. The data represent the numbers of live mice at the indicated day postinfection.

In the causal prophylactic rodent malaria model, mice are intravenously infected with P. berghei sporozoites that target the liver. After an incubation period of 48 h, the P. berghei liver schizonts will release merozoites that initiate blood-stage infection and cause symptoms of disease within 5 or 6 days. In this model, a single oral dose of 10 mg of KAF156/kg administered 2 h before infection was fully protective (Fig. 3) (14).

### KAF156 inhibits gametocytogenesis and blocks transmission to the Anopheles mosquito.

**FIG 4:**
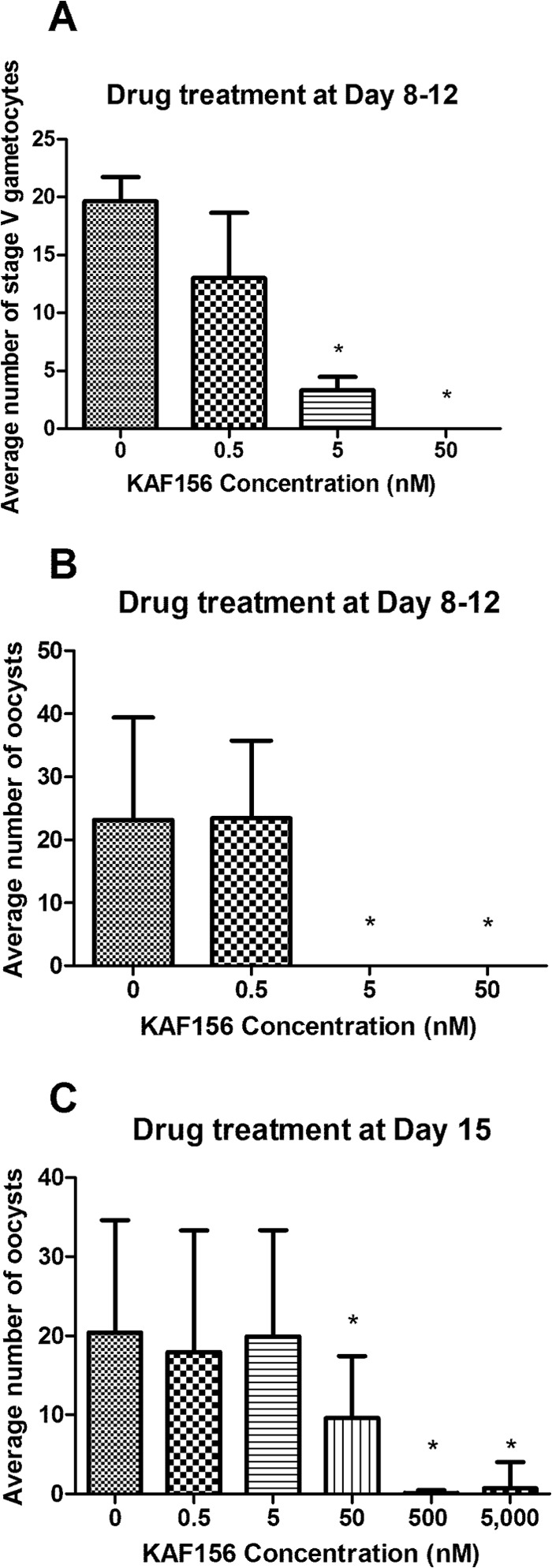
KAF156 inhibits gametocyte development and blocks parasite transmission to mosquitoes. Three (A and B) or two (C) independent experiments were carried out, and the mean values are reported on bar graphs with the standard error of the mean indicated. (A) Cultures of immature stage II gametocytes were treated with various concentrations of KAF156 on days 8 to 12 after the induction of gametocytogenesis. The negative control (KAF156 concentration = 0 nM) cultures were treated with DMSO (vehicle) at a 0.1% final concentration. The total number of mature stage V gametocytes (males and females) per 5,000 erythrocytes was assessed by microscopy on day 13 for each experiment. (B) An SMFA was used to evaluate the transmission potential of parasites cultures treated on days 8 to 12. (C) Viable and fully mature stage V gametocytes (males and females) were treated with KAF156 on day 15 after induction and fed through SMFA to mosquitoes. For both panels B and C, no toxicity to mosquitoes was observed at any of the compound concentrations, and the number of oocysts in the midgut of an infected mosquito was counted for at least 20 infected mosquitoes for each independent experiment. An asterisk (*) indicates that the mean is significantly different (*P* < 0.05) from the untreated control in a one-way analysis of variance, followed by a Dunnett's multiple-comparison test.

The completion of the sexual phase of the Plasmodium life cycle in infected red blood cells yields fully mature stage V female and male gametocytes that are transmitted to the Anopheles mosquito. We evaluated the activity of KAF156 against gametocyte formation and their transmission to the mosquito vector. Early-stage gametocytes treated with KAF156 on days 8 to 12 after the induction of gametocytogenesis showed a significant dose-dependent reduction in the total number of stage V gametocytes (Fig. 4A). These data suggest that the compound is a potent inhibitor of gametocyte maturation *in vitro* at concentrations as low as 50 nM. Consistent with this observation, when fed to mosquitoes through a standard membrane-feeding assay (SMFA) (23), all three cultures treated with 5 nM KAF156 yielded zero oocysts (Fig. 4B). Taken together, the data suggest that KAF156 has a profound effect on the final and critical steps of gametocyte maturation exflagellation. This range of activity against Plasmodium sexual stages is at least comparable to, if not superior to, what has been reported before with approved antimalarial drugs (32, 33).

We also evaluated KAF156 transmission blocking potential. Viable and fully mature stage V gametocytes were incubated for 15 min with various KAF156 concentrations and fed to mosquitoes through an SMFA. In this assay, KAF156 showed a clear dose-dependent effect with >90% reduction of oocyst numbers at a concentration of 500 nM (Fig. 4C). Notably, the compound had no adverse effects on mosquito viability. We then confirmed these observations *in vivo* in a P. berghei rodent malaria model. Infected mice treated with a single dose oral of KAF156 at 100 mg/kg were found to be not infectious to Anopheles mosquitoes feeding on their blood (see Fig. S2 in the supplemental material). Taken together, our results demonstrate that KAF156 inhibits the maturation of P. falciparum sexual stages and effectively blocks parasite transmission to Anopheles mosquitoes.

## DISCUSSION

Cell-based screening is a well-established method for the discovery of new anti-infective drugs and has been historically successful for antimalarial drug discovery (34). Given the large number of novel antimalarial scaffolds recently discovered that target asexual blood stages (35–38), identifying compounds in these sets that are also active on liver stages and mature gametocytes will allow for the discovery of next-generation antimalarials, compatible with the SERCaP target product profile. Indeed, there are several recent reports of phenotypic assays suitable for screening compounds for activity against liver schizonts (13, 39), gametocytes (32, 40), and possibly hypnozoites (41, 42).

We show here that the clinical candidate KAF156, optimized for its potent blood-stage activity, is also fully protective in a liver-stage mouse model of malaria and blocks disease transmission. Screening of asexual erythrocytic actives with orthogonal assays for sexual and liver stages has greatly facilitated next-generation antimalarial drug discovery. Currently, very few drug targets have been chemically validated across the parasite life cycle (43–45). It will thus be important to apply these screens to all available asexual blood-stage hits since the identification of compounds active across multiple stages will help define the parasite genome relevant for the discovery of next-generation antimalarial drugs. Indeed, numerous postgenomic tools are now available to identify the molecular targets of novel antimalarials (46). The overlap of the parasite proteome between all stages is predicted to be small (47–49). This may be because only the most abundant proteins are detected by full genome proteomic methods.

The discovery of the imidazolopiperazines is thus significant, since the determination of their mechanism of action and their molecular target would provide opportunities to rationally screen for and design compounds targeting sexual and hepatic stages. In a first step toward the deconvolution of this series' mechanism of action, we selected for drug-resistant parasites *in vitro* and identified nonsynonymous SNPs in *pfcarl*, which encodes a protein of unknown function with seven putative transmembrane domains (see Fig. S1 in the supplemental material) (13). It is worth noting that the PfCARL protein is expressed during asexual and sexual blood stages (49, 50). Previously reported expression studies also indicate that the PfCARL ortholog in P. berghei is expressed in gametocytes, as well as in the blood and liver stages (47). Mutations in PfCARL are specific to imidazolopiperazines and do not result in resistance to other classes of drugs (see Table S3 in the supplemental material). It is thus tempting to speculate that PfCARL is the direct molecular target of KAF156; however, we cannot currently rule out that PfCARL may act as a transporter for imidazolopiperazine. This remains to be determined with further experiments aiming to elucidate the function of the *pfcarl* gene product.

Consistent with our inability to raise drug-resistant parasites with the African FCR3 clone and the moderate frequency of KAF156 drug-resistant mutations in the Southeast Asian Dd2 clone, none of the SNPs identified have been previously reported in clinical isolates (PFC0970w search at http://plasmodb.org). This suggests that the KAF156 drug-resistant mutations are rare and that the amino acids involved are functionally important. Nonetheless, our data show that significant resistance can be acquired with a few SNPs. Since this compound is moving toward clinical development, the identification of a suitable drug partner will be crucial, and it will be important to monitor the possible emergence of mutations in the *pfcarl* locus and particularly at position 1076. Indeed, the set of SNPs reported here will be valuable to assess the drug resistance liability and help devise a drug combination strategy to minimize this risk.

KAF156 has favorable drug-like properties with pharmacokinetics in preclinical species compatible with once-daily dosing and no significant *in vitro* safety liabilities (14). Recently, the compound went through an extensive Good Laboratory Practices safety and pharmacological preclinical assessment that supported progression to human clinical trials. If KAF156 is shown to be safe and effective, it could be the first new antimalarial drug combining potent prophylactic, therapeutic, and transmission-blocking activities—a significant addition to the armamentarium for the malaria eradication agenda.

## Supplementary Material

Supplemental material
