# Supplementary material for: KAF156 Is an Antimalarial Clinical Candidate with Potential for Use in Prophylaxis, Treatment, and Prevention of Disease Transmission
Source: Antimicrob Agents Chemother. 2014 Sep;58(9):5060–7. doi: 10.1128/AAC.02727-13 (PMC4135840; doi:10.1128/AAC.02727-13)
Supplement: Supplemental material [file supp_58_9_5060__index.html]

KAF156 Is an Antimalarial Clinical Candidate with Potential for Use in Prophylaxis, Treatment, and Prevention of Disease Transmission — Supplemental material 

# KAF156 Is an Antimalarial Clinical Candidate with Potential for Use in Prophylaxis, Treatment, and Prevention of Disease Transmission

## Supplemental material

**Files in this Data Supplement:**

- Supplemental file 1 -

  Tables S1 to S4 and Fig. S1 and S2.

  PDF, 197K
