# Supplementary material for: KAF156 Is an Antimalarial Clinical Candidate with Potential for Use in Prophylaxis, Treatment, and Prevention of Disease Transmission
Source: Antimicrob Agents Chemother. 2014 Sep;58(9):5060–7. doi: 10.1128/AAC.02727-13 (PMC4135840; doi:10.1128/AAC.02727-13)
Supplement: Supplemental material [file AAC.02727-13_zac008143170so1.pdf]

Supplemental Material

TABLE S1 KAF156 is highly potent against *P. falciparum*.

P. falciparum strain	Drug Resistance	KAF156 IC_{50} (nM)
3D7	SFX	9.0 ± 1.0
W2	CQ, PYR, QN, SFX	7.7 ± 0.7
Dd2	MEF, CQ, PYR	7.1 ± 1.7
HB3	PYR	16.6 ± 2.2
FCB	CQ	6.4 ± 0.4
Camp R	PYR	17.4 ± 5.8
D10	None known	12.1 ± 9.7
D6	None known	10.2 ± 1.7
K1	CQ, PYR, SFX	7.3 ± 0.7
NF54	None known	16.4 ± 3.7
TM91C235	PYR	11.4 ± 0.7
3BAG	CQ, PYR	9.1 ± 1.3
C188	PYR	7.5 ± 0.7
FCR3	CG, CQ, PYR	8.4 ± 1.4
7G8	CG, CQ	6.0 ± 0.3

KAF156 exerts low nanomolar potency against a panel of drug susceptible and drug-resistant *P. falciparum* strains. CG, cycloguanil; CQ, chloroquine; MEF, mefloquine; PYR, pyrimethamine; QN, quinine; SFX, sulfadoxine. Data shown as median ± SD based on at least 6 independent experiments.

TABLE S2 *In vivo* effective doses of KAF156 in a *P. berghei* experimental mouse model

Compound	ED_{50} (mg/kg)	ED_{90} (mg/kg)	ED_{99} (mg/kg)
Chloroquine*	1.9	4.2	8.4
Mefloquine*	3.8	5.2	6.6
Artesunate*	5.9	20.5	57.5
KAF156[†]	0.6	0.9	1.4

Experimental doses resulting in a 50%, 90% and 99% decrease in parasitemia relative to control values were determined for KAF156 and standard antimalarials. Values were determined after treatment of 5 mice with a single dose 24 hours after infection with *P. berghei* ANKA blood-stage parasites. Parasitemia was determined by microscopy on day 3 (72 hours after infection) to determine effective dose values. Data are shown as means calculated from at least two independent experiments. * T/A (Ethanol/Tween 80/water 3/7/90); [†] 5% Solutol HS15; GFP-ANKA was used in the reference study with chloroquine, mefloquine, and artesunate and parasitemia was determined by FACS analysis

TABLE S3 KAF156 resistance mechanism is unique and standard drugs do not show cross resistance.

Selection Agent	Culture #	SNP* (*Pfcarl*)	Artemisinin	Mefloquine
			IC_{50} (nM)	IC_{50} (nM)
DMSO		None (Dd2, WT)	13 ± 7.9	17 ± 9.1
KAF156	1AR	Q821H, S1076R	11 ± 1.9	5.6 ± 5.2
	1BR	P822T, S1076R	7.3 ± 3.8	9.0 ± 6.2
	2BR	S1076I	16 ± 3.0	7.0 ± 6.0
	3AR	E834D	7.6 ± 2.9	7.6 ± 5.5
	3BR	E834D	6.3 ± 3.3	7.8 ± 5.3

Five independent cultures of *P. falciparum* strain Dd2 were cultured in the presence of increasing concentrations of KAF156 over 4 months. Parasitemia was monitored daily and compound concentration was increased 2-fold when the parasitemia reached ≥3%. For each of the resistant strains the SNPs detected and antimalarial activity (IC_{50}) is indicated for artemisinin and mefloquine (data are shown as median ± SD for 4 or more independent experiments). Strain Dd2 is resistant to pyrimethamine (IC_{50} >12.5 μM). *Single nucleotide polymorphisms (SNPs) were confirmed by capillary sequencing of *Pfcarl* (PFC0970w).

TABLE S4 Frequency of resistance to KAF156.

Strain	Starting inoculum / flask	Resistance acquisition	Parasitemia >1%
Dd2	1 x 10^6 parasites	0 / 3 cultures	-
Dd2	1 x 10^7 parasites	1 / 3 cultures*	Day 23
Dd2	1 x 10^8 parasites	3 / 3 cultures	Day 18 - 25
Dd2	4 x 10^8 parasites	3 / 3 cultures	Day 17 - 18
Dd2	6.8 x 10^8 parasites	3 / 3 cultures	Day 25 - 30
Dd2	1.3 x 10^9 parasites	3 / 3 cultures	Day 17
Dd2	4 x 10^9 parasites	3 / 3 cultures	Day 18
FCR3	5.7 x 10^8 parasites	0 / 3 cultures	-

P. falciparum cultures of either Dd2 or FCR3 strains were seeded at various starting parasite loads (in triplicate) and exposed to 40 nM KAF156 continuously for up to 60 days. The emergence of parasites was detected by Giemsa smear and the time required to reach >1% parasitemia was noted. * For selections starting with 1 x 10^7 parasites, one positive culture was obtained, however these parasites did not display a shift in KAF156 IC_{50} relative to the parent strain and did not possess SNPs in *pfcarl*.

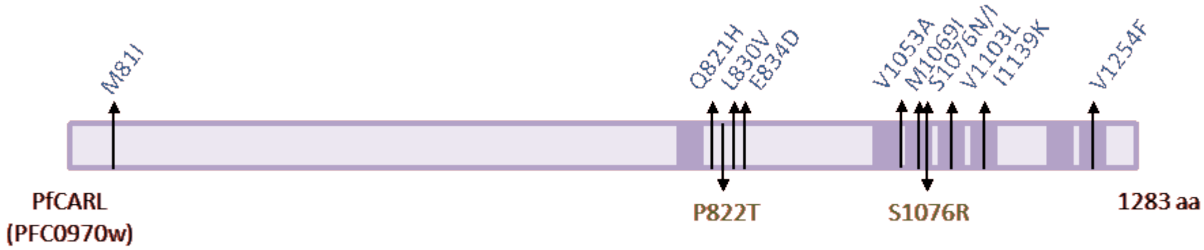

FIG S1 SNPs identified in *pfcarl* by whole genome sequencing, capillary sequencing or tiling array analysis (1). Schematic of *pfcarl* showing the transmembrane domain. SNPs identified in (1) are shown above the schematic, novel SNPs identified in KAF156-resistant clones are shown below.

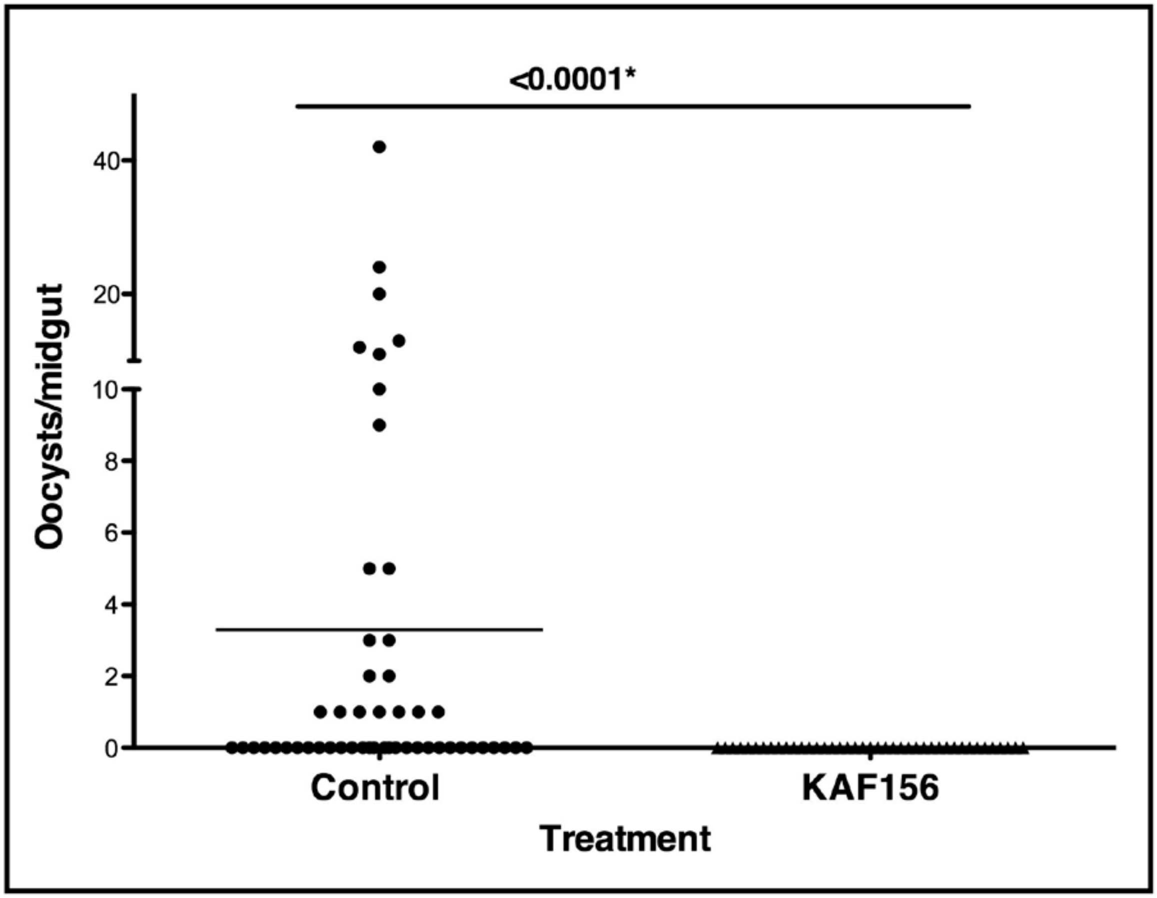

FIG S2 KAF156 blocks parasite transmission in a rodent malaria model. Number of oocysts found in the midguts of 41 mosquitoes fed on 2 mice treated with a single oral dose of KAF156 at 100 mg/kg (▲ closed triangles) and 51 mosquitoes fed on 2 untreated mice (● closed circles).* Two-tailed P-value determined in a non-parametric Mann-Whitney test (95% confidence limit).

References

1. **Meister, S, Plouffe, DM, Kuhen, KL, Bonamy, GM, Wu, T, Barnes, SW, Bopp, SE, Borboa, R, Bright, AT, Che, J, Cohen, S, Dharia, NV, Gagaring, K, Gettayacamin, M, Gordon, P, Groessl, T, Kato, N, Lee, MC, McNamara, CW, Fidock, DA, Nagle, A, Nam, TG, Richmond, W, Roland, J, Rottmann, M, Zhou, B, Froissard, P, Glynne, RJ, Mazier, D, Sattabongkot, J, Schultz, PG, Tuntland, T, Walker, JR, Zhou, Y, Chatterjee, A, Diagana, TT , Winzeler, EA**. 2011. Imaging of *Plasmodium* liver stages to drive next-generation antimalarial drug discovery. Science 334: 1372-1377
